# Adults with Dyslexia Demonstrate Large Effects of Crowding and Detrimental Effects of Distractors in a Visual Tilt Discrimination Task

**DOI:** 10.1371/journal.pone.0106191

**Published:** 2014-09-03

**Authors:** Rizan Cassim, Joel B. Talcott, Elisabeth Moores

**Affiliations:** School of Life and Health Sciences, Aston University, Birmingham, West Midlands, United Kingdom; University of British Columbia, Canada

## Abstract

Previous research has shown that adults with dyslexia (AwD) are disproportionately impacted by close spacing of stimuli and increased numbers of distractors in a visual search task compared to controls [1]. Using an orientation discrimination task, the present study extended these findings to show that even in conditions where target search was not required: (i) AwD had detrimental effects of both crowding and increased numbers of distractors; (ii) AwD had more pronounced difficulty with distractor exclusion in the left visual field and (iii) measures of crowding and distractor exclusion correlated significantly with literacy measures. Furthermore, such difficulties were not accounted for by the presence of covarying symptoms of ADHD in the participant groups. These findings provide further evidence to suggest that the ability to exclude distracting stimuli likely contributes to the reported visual attention difficulties in AwD and to the aetiology of literacy difficulties. The pattern of results is consistent with weaker and asymmetric attention in AwD.

## Introduction

Although phonological difficulties are well-established as core features underlying literacy impairments [2]–[4], focus is being increasingly devoted to the role of visual attention difficulties in reading disorders, including developmental dyslexia. A variety of attention difficulties have been reported in dyslexia, including asymmetric attention distribution [5], reduced visual attention span [6], increased crowding effects [1], [7]–[11], difficulty in using cues [12]–[14] and less effective mechanisms for noise exclusion [15], [16]. Controversially, it has been argued that even core phonological language difficulties shown in dyslexia can be statistically accounted for by an underlying visual deficit [17]. The majority of research implicates a model of dyslexia based on multiple underlying deficits [18], with attention difficulties as an additional contributing factor to the genesis of reading impairments in some individuals. Moreover, the high comorbidity between dyslexia and attention deficit hyperactivity disorder (ADHD) [19] may also account for some of the covariance between deficits in visual attention and core reading skills. Here, we examined three specific aspects of visual attention: (i) visual crowding, (ii) the ability to exclude distractors and (iii) the distribution of attention across the visual field. We also investigated the relationship of these three aspects of attention with four different measures of literacy and with the presence of sub-clinical ADHD symptoms, in our sample.

### 1.1 Effects of visual crowding

Visual crowding occurs when stimuli become more difficult to either detect or discriminate when surrounded by other stimuli, compared to when they are presented in isolation. Crowding is a phenomenon that occurs in nearly every visual context. It can occur with simple stimuli - such as orientation gratings – and also with complex stimuli such as letters [20]. Efficient allocation and control of visual attention can ameliorate the negative effects of crowding [21], [22], although Dakin, Bex, Cass and Watt [23] have argued that crowding does not specifically reflect a limitation in attention. He, Cavanagh and Intriligator have provided alternative evidence from an orientation adaptation and discrimination task that suggests that it is attention – rather than visual acuity – that ultimately limits spatial resolution under normal circumstances [24]. Several studies have suggested that persons with reading impairments such as dyslexia suffer more from crowding than do similarly aged control readers [1], [7]–[11], [25]. Research in this area, however, has mainly used letter or letter-like stimuli to investigate this hypothesis. Because dyslexia is also associated with deficits in recognition and processing of linguistic stimuli – including letters – it is difficult to adjudicate between effects linked to visual attention and those associated with processing the symbols of language in such tasks.

### 1.2 Noise (distractor) exclusion

In addition to evidence for increased crowding effects in dyslexia, other studies also identify difficulties in excluding distracting stimuli. Sperling *et al.* [15], [16] showed that performance of adults in a visual motion detection task only correlated with their reading ability in conditions where the signal to noise ratio was low. Using a visual search paradigm, Roach and Hogben [26] measured psychophysical thresholds of adults with dyslexia (AwD) and controls for detection of a tilted target stimulus presented amongst vertical distractors. Although the set size effect of the control group, but not the AwD, was diminished when targets were cued, they provided evidence [13], [14] to suggest that the difficulty of the AwD was one of ineffective noise exclusion, rather than problems detecting and localising the cue *per se*. Using a similar paradigm, Moores *et al.* [1] also reported that AwD could use pre-cues to modulate attention, but that they were less successful at using them to counter effects of increasing numbers of distractors on target detectability. A limitation of these studies, however, is that visual search paradigms were used, in which - for uncued conditions - the target location was not known. Therefore, any potential beneficial effects of cueing for either group could reflect reduced spatial uncertainty regarding target location, rather than the effects of enhanced attention or distractor exclusion. As an alternative, the current study presented the target stimulus at one of only two possible fixed locations in the left and right visual fields. This manipulation minimised the potential for eye movements towards a fixed stimulus location. Also, because previous research has shown an asymmetrical distribution of attention in dyslexia, it allowed us to replicate this effect here.

### 1.3 Distribution of attention

Facoetti, Paganoni and Lorusso [27] have previously reported that a group of children with dyslexia (CwD) showed more distributed or diffuse attentional focus, evidenced by a relatively flat profile of reaction times for stimulus detection with increasing retinal eccentricity. Facoetti and Molteni [28] demonstrated that this profile only occurred for stimuli presented in the right visual field, whereas responses of CwD in the left visual field were slower than those of controls overall, but were faster for stimuli presented at central compared to peripheral locations. They proposed that an attention disorder affecting the left visual field would explain the slower responses overall, with the lack of a performance gradient across eccentricity accounting for the over-distractibility associated with stimulus processing in the right visual field. Hari, Renvall and Tanskanen [29] also reported slower performance in AwD in the left visual field on two psychophysical tasks and from this evidence proposed a left-sided ‘minineglect’ in dyslexia. Consistent with this hypothesis, Waldie and Hausmann [30] reported a reversal of the normal leftward bias observed in a line bisection task in CwD and in children with ADHD (see also [31]). Further evidence for an asymmetry was provided by Facoetti and Turatto [5], who reported a reduced effect of flankers in the left visual field, and by Facoetti, Turatto, Lorusso and Mascetti [32], who showed slower reaction times to invalidly cued targets in the left compared to the right visual field. A body of psychophysical evidence therefore points toward an asymmetric and more diffuse distribution of attention in dyslexic readers.

### 1.4 Relationship between literacy and effects of crowding, set-size and visual field asymmetry

It is important to determine whether the differences in attention associated with dyslexia are related to measures of literacy, or more to third variables which also co-occur with dyslexia and other development disorders. Relationships between measures of visual attention and of literacy have previously been demonstrated. Sperling *et al.* [16] reported a moderate [33] correlation between reading ability and ability to detect visual coherent motion amongst noise in adults. Moores *et al.* [1] reported moderate to strong correlations between literacy measures and dependence on attentional cues, effects of crowding and the impact of a greater number of distractors. Facoetti *et al.* [34] reported a strong correlation in dyslexic readers between nonword reading accuracy and the extent of a deficit in attentional inhibition in the right visual field (i.e. the finding that when cued to the left visual field, targets in the right visual field are not inhibited). All these associations between reading and attention variables, however, might plausibly be related to the overlapping dimensions dyslexia shares with ADHD, rather than to literacy skills directly. In the present study, we examined the relationships between four measures of literacy (word reading accuracy, word spelling accuracy, real word reading efficiency and nonword reading efficiency) and our measures of crowding, distractor exclusion and attention asymmetry.

### 1.5 Associations with ADHD

Previous research has suggested that dyslexic readers suffer more from crowding, are less effective at excluding distractors, and have a different distribution of attention compared to controls. Furthermore, these difficulties correlate with literacy abilities. However, it has been estimated that upwards from 15% of children with dyslexia also have co-occurring attention deficit hyperactivity disorder (ADHD) and around 36% of children with ADHD are estimated to have dyslexia [19]. According to the Diagnostic and Statistical Manual of Mental Disorders 5^th^ Edition [35] the primary symptoms of ADHD are ‘inattention’ (which includes poor sustained attention, forgetfulness and distractibility) and ‘hyperactivity/ impulsivity’. Most contemporary theories of ADHD argue that the core deficits are associated with either poor inhibitory control [36] or aversion to delay [37]. However, an alternative explanation may be that reported ‘attention’ difficulties in dyslexia may be accounted for by concomitant ADHD symptoms. The dimensions of literacy and attention are continuous variables, with categories derived from rather arbitrary statistical cut-offs. Although many dyslexia studies investigating aspects of visual attention have excluded participants with actual diagnoses of ADHD from their studies, few have specifically investigated the potential role of sub-clinical ADHD symptoms by controlling statistically for their presence. It should be noted, however, that ‘inattention’ type symptoms as discussed in the context of ADHD may be conceptually different from the type of visual selective attention difficulties investigated in this study and other research in this area. A lack of sustained attention or a general distractibility would be expected to adversely affect performance across *all* conditions of any task, whereas research into visual attention difficulties in dyslexia invariably reports dissociated patterns of deficit which only present in particular conditions; for example only when a display is crowded, or only in the left visual field. Nonetheless, in a different context, it has been shown that ADHD symptoms can mediate the effects of differences in performance variables between groups of persons with dyslexia and controls [38], so in the current study it was important to assess the potential impact of sub-clinical ADHD effects as an alternative hypothesis.

### 1.6 Limitations, summary and overview

Moores *et al.* [1] showed that a greater dependence on pre-cues, larger effects of crowding and the impact of increased numbers of distractors all correlated strongly with measures of literacy skill. However, as discussed in *section 1.2*, this task was a visual search task in which the target location was not known (unless pre-cued). The advantage of pre-cueing in this task could therefore have been due to a reduced spatial uncertainty over target location as well as – or instead of – enhanced attention and/ or noise exclusion. In addition, crowding could have simply made the target more difficult to locate, rather than more difficult to discriminate *per se* [39]. Furthermore, our previous study did not investigate the potential role of ADHD symptomatology on task performance. With any potential effects of ADHD statistically removed, the present study therefore investigated whether: (i) AwD experienced increased crowding effects and difficulty excluding distractors even when target location was known, (ii) any increased difficulties in excluding distractors or in crowding showed any asymmetry in AwD and (iii) any difficulties with crowding, excluding distractors, or any asymmetry had any relationship with four different measures of literacy.

## Materials and Methods

### 2.1 Participants

Participants provided informed written consent which conformed to the procedures approved by the Aston University's Ethics Committee on use of human participants. The study was approved by Aston University Ethics Committee. Sixteen control adults (7 males) and eighteen AwD (8 males) equated for age and full scale IQ took part in this study (Eleven control adults (7 males) and thireteen AwD (6 males) also took part in our previous study [1]). Participants were also asked to provide ratings of their behaviour across the dimensions of inattention and hyperactivity/impulsivity on the Barkley Current Symptoms Scales [40]. Scores of greater than 6 on either measure, or exceeding 9 for the sum of inattention and hyperactivity scores, are indicative of dimensions in which further exploration toward a clinical diagnosis of ADHD is usually suggested [40]. The selection criteria required that each participant had a profile of enduring reading and spelling difficulties and/or previous clinical diagnosis, but no prior history of any other developmental disorder. The control group was required to have no previously reported problems in spelling or reading. All participants were required to have a minimum full scale IQ of 90, English as their first language and normal or corrected-to-normal vision. All participants also had completed at least some higher education (on average 12.8 years of schooling, SD = 0.49) and most of them (15 controls and 15 AwD) were either previously or currently enrolled in university degree programmes.

Participants from both groups were initially screened using a battery of assessments for cognitive skills and literacy achievement. All AwD provided a recent report from an educational psychologist at the time of testing, which provided the estimate of full-scale IQ used in the study (using the Wechsler Adult Intelligence Scale (WAIS) [41]). The IQ test for control participants was administered on the day of testing (using the Wechsler Abbreviated Scale of Intelligence (WASI) [42]), unless they had been previously assessed with either the Wechsler Adult Intelligence Scale or the Wechsler Abbreviated Scale of Intelligence. Reading and spelling achievement was measured using the Wechsler Individual Achievement Test (WIAT-II UK) reading and spelling subtests [43] unless these measures had already been administered within the previous 12 months. The Test of Word Reading Efficiency (TOWRE) [44] - a speeded reading test designed to measure word reading accuracy and fluency - was also conducted. The Sight Word Efficiency (SWE) test provided a fluency measure for real words, whilst the Phonemic Decoding Efficiency (PDE) test provided a decoding measure for accuracy and fluency of pronounceable non-words.

A comparison of demographic and psychometric data for both the controls and AwD is summarised in Table 1. Independent sample t-tests (with Levene's correction for unequal variances when appropriate) showed no significant differences between groups for age [*t (28.08)* = 0.46], level of education [*t (22.14)* = 1.27] or IQ [*t (31.98)* = 2.51]. The average performance of the AwD group was significantly lower than the controls for all measures of literacy: WIAT-II word reading [*t (31.53)* = 9.54, *p*<.001], WIAT-II spelling [*t (28.26)* = 6.11, *p*<.001], TOWRE SWE [*t (32.00)* = 3.79, *p*<.001] and TOWRE PDE [*t (28.06)* = 19.46, *p*<.001]. AwD also had higher scores on the ADHD measure [*t (29.62)* = −3.87, *p*<.001].

**Table 1:** Demographic and psychometric group data.

	Control (n = 16)		AwD (n = 18)		*p*-value
	Mean	SD	Mean	SD	
**Age (years)**	26.69	5.65	25.89	4.35	ns
**Education** a	12.94	0.25	12.72	0.67	ns
**Full-Scale IQ**	124.19	6.53	118.11	7.58	ns
**Spelling (WIAT-II UK)** b	116.50	5.39	100.72	9.25	<.001
**Reading (WIAT-II UK)** b	110.31	3.18	98.44	4.06	<.001
**TOWRE (SWE)** b	107.69	6.09	99.28	6.86	<.001
**TOWRE (PDE)** b	116.63	3.44	95.94	2.65	<.001
**ADHD**	1.44	1.15	3.39	1.75	<.05

a The level of education represents years of schooling from year 1 (infant school) to year 13 (college/sixth form).

b The composite standard scores (SS). For the TOWRE these were calculated using the norms 17∶0–24∶11 (years∶ months).

### 2.2 Stimuli and apparatus

The tilt discrimination experiment was developed using E-Prime Version 2-Professional [45] and conducted on a P4 Dell Optiplex GX 260 desktop computer displaying the output on a 19-inch CRT Vision Master Pro 510 monitor (1024×768-pixel screen resolution, 60-Hz refresh rate).

The stimuli consisted of five greyscale sine-phase Gabor patches generated using Matlab (MathWorks Ltd) comprising wavelength (*λ* = 10 pixels per cycle) and a Gaussian standard deviation (*σ* = 10). The target Gabors were tilted (*θ*) at different angles relative to the vertical distractors in two conditions which varied in difficulty: i) *θ* = ±5° (‘easy’ condition) and, ii) *θ* = ±2° (‘difficult’ condition).

The participants were asked to support their head within a chin rest while seated at a viewing distance of 57 cm in a dimly lit room. Participant responses were entered using specific key presses on a standard computer keyboard.

### 2.3 Design

The independent variables of interest were group (AwD or Control), display type (zero distractors, two-spaced distractors, two-crowded distractors, four distractors or eight distractors), task difficulty (easy or difficult tilt) and visual field (VF: left or right). The direction of tilt was also randomised. The dependent variable was percent correct for the detection of the correct orientation of the target stimulus.

**Figure 1:**
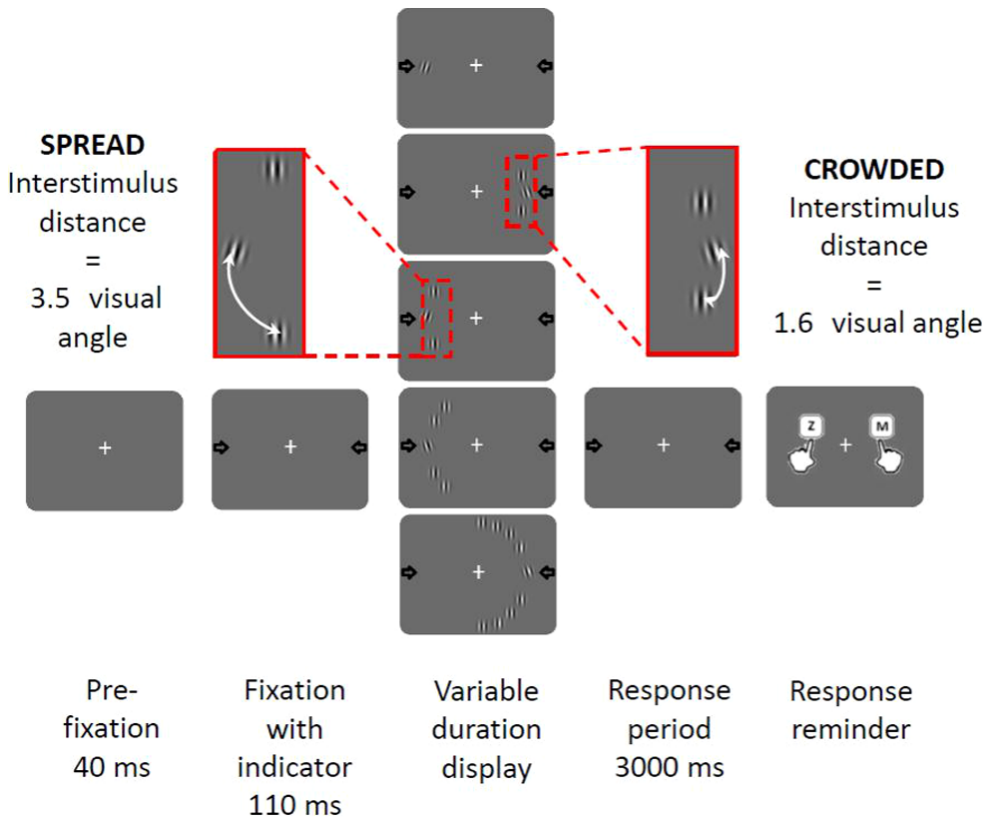
Schematic representation of the stimulus sequence for the tilt discrimination task. The two possible target locations (left and right sides of the screen) were indicated on the display screen with arrows. Targets could be presented either alone, or surrounded by two, four or eight distractors arranged symmetrically above and below the target. In conditions in which two distractors were presented they could be presented with an interstimulus distance of either 3.5 degrees (spread) or 1.6 degrees (crowded). Participants responded whether the target tilted left or right using the z and m keys on the computer keyboard.

Participants performed a two alternative forced choice task (see Figure 1) in which they were required to indicate the orientation (left or right tilt) of a single tilted target stimulus that was always presented in the centre of a variable length string of vertically oriented distractors. On a given trial, there was an equal probability of the target stimulus having a tilt of either ±2° (easy) or ±5° (difficult). The string was comprised of varying configurations presented with equal probability: zero distractors, two crowded distractors, two spread distractors, four distractors or eight distractors. The stimuli (target and distractors) were positioned either to the left-VF or right-VF (50% probability) of the display on the circumference of an imaginary semi-circle located 5° of visual angle peripheral to the central fixation point. In either VF, the target stimulus always appeared at a fixed central position (indicated by the arrow placeholder as in Figure 1) whilst the distractors were arranged symmetrically above and below it in the same hemifield. To evaluate the effect of distractors alone, crowding effects in the different array conditions were kept constant by separating the distractors closest to the target by an interstimulus distance of 3.5° visual angle from the target while the distance between distractors was constant throughout at 1.6° visual angle. The exception to this was in the two distractor crowded condition in which the effects of crowding were systematically manipulated by positioning the two distractors nearer to the target (target-distractor separation of 1.6° visual angle). Although our spacing manipulation led to unequal spacing of stimuli in four and eight distractor conditions, it enabled us to manipulate the number of distractors presented in a single hemifield *without* varying the spacing between the central target and its nearest distractors (i.e. without varying the degree of crowding of the target). Stimuli were presented with consistent spacing in the two conditions where we specifically investigated crowding effects (since only two distractors were used in each case).

### 2.4 Procedure

Initiated by a single key press, each trial sequence commenced with an onset of a blank grey screen with a central fixation point (**+**) of 40 ms duration. Participants were instructed to visually fixate this position throughout the entire duration of each trial. A second fixation screen followed for 110 ms which had arrows on each side of the display corresponding to the two specific locations at which the target could appear. This was followed by a variable duration display (containing any one of the five display types) and then by a further fixation screen until response (see Figure 1 for a visual schematic of the experimental set-up). If a response was not made a reminder screen followed after 3000 ms.

The display was of a variable duration titrated to achieve individual accuracy levels of between 60% and 90%. The experimental session had a total of 15 blocks with each block having 40 trials (total of 600 trials). In each block, two trials (one target tilting right and one left) of each of 20 conditions were conducted and the detection accuracy calculated for that block. When overall response accuracy either surpassed 90% or fell below 60% a 10 ms reduction or increase (respectively) was made to the stimulus duration. Before commencing the main experiment, the participants performed two blocks of practice and calibration sessions (20 trials each) to ensure that they were sufficiently familiar with the procedure and to establish an accuracy level within the 60%–90% range. The average display durations of the AwD and the control group did differ significantly (119 ms vs. 98 ms, *t(32)* = 1.47, *p<.05*).

## Results

The results comprised the proportion of correct discriminations in each of the 20 conditions and are available in the Dataset S1. We first investigated effects of visual crowding using ADHD score as a covariate. Second, we investigated the effects of increasing distractor set size and visual field, again using ADHD as a covariate. Third, we examined the relationship between literacy measures and measures of crowding, distractor set-size and asymmetry of attention with statistical effects of ADHD partialled out from the correlations.

### 3.1 Effects of Crowding

To assess the potential effects of crowding, a four-way ANCOVA was conducted using the variables: group (controls, AwD); set-size-two display type (spread, crowded); task difficulty (easy, hard) and visual field (left, right). Analyses indicated a significant main effect of group (*F*_(1,31)_ = 43.77, *p*<.001, *η* ^2^_p_ = .59) with higher performance in controls. There were also significant main effects of display type (*F*_(1,31)_ = 6.46, *p*<.05, *η* ^2^_p_ = .17) and task difficulty (*F*_(1,31)_ = 13.02, *p*<.001, *η* ^2^_p_ = .30), showing inferior performance in crowded and difficult conditions. The effect of visual field was not significant (*F*_(1,31)_ = 2.46). ADHD was not a significant covariate (*F*_(1,31)_ = 0.69). Significant interactions between display type and group (*F*_(1,31)_ = 8.67, *p*<.01, *η* ^2^_p_ = .22) and task difficulty and group (*F*_(1,31)_ = 10.66, *p*<.01, *η* ^2^_p_ = .26) suggested a different pattern of performance by AwD when the discrimination task was difficult and the display was crowded. No other interactions reached statistical significance.

**Figure 2:**
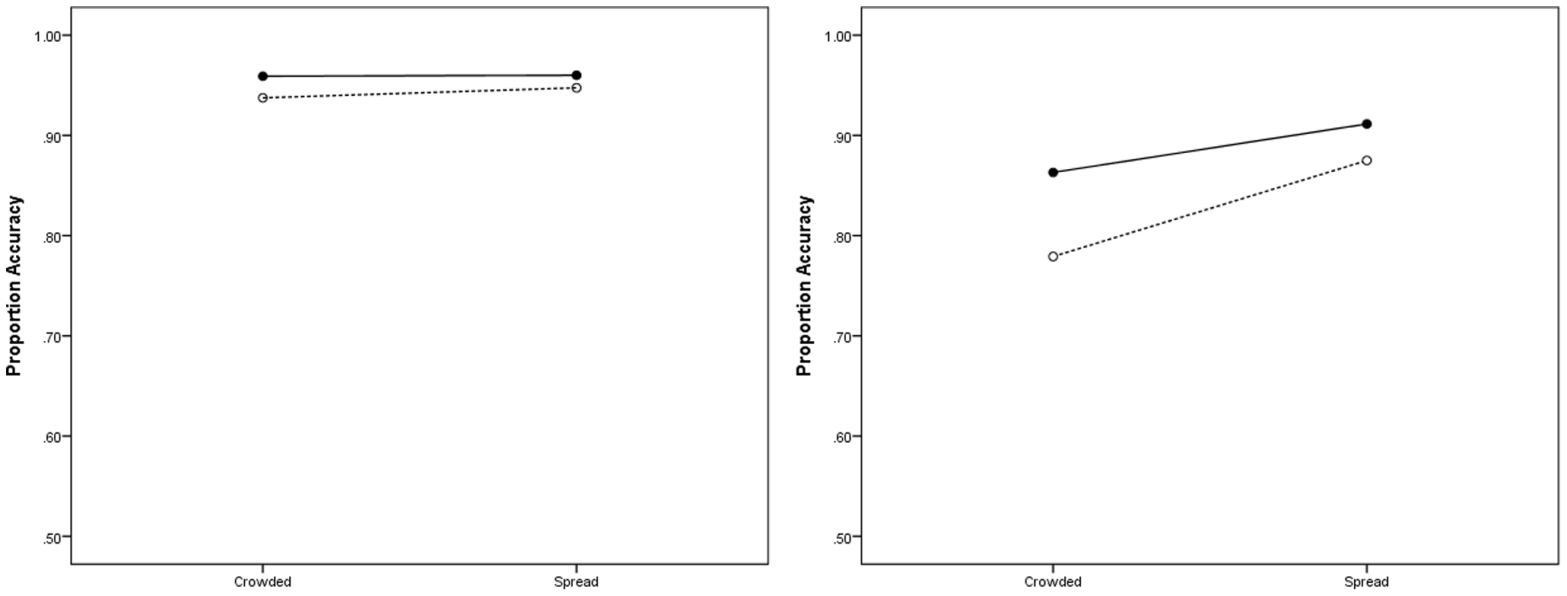
Interaction plots indicating performance accuracy for both controls (left panel) and AwD (right panel) plotted as a function of display type (crowded vs. spread) and task difficulty (solid lines - easy conditions and dotted lines - hard conditions).

To investigate the significant interactions, analyses were conducted on each group separately with ADHD as a covariate. The descriptive statistics are summarised graphically in Figure 2. A significant effect of display type emerged for AwD (*F*_(1,16)_ = 14.18, *p*<.001, *η* ^2^_p_ = .47) but not for controls (*F*_(1,14)_ = .108). Both the control group (*F*_(1,14)_ = 9.18, *p*<.05, *η* ^2^_p_ = .40) and the AwD (*F*_(1,16)_ = 6.97, *p*<.05, *η* ^2^_p_ = .30) showed a significant effect of task difficulty. ADHD was not a significant covariate for either the controls (*F*_(1,14)_ = .001) or the AwD (*F*_(1,16)_ = 1.05). The main effect of visual field was not significant for either AwD or controls (Fs<1), although in controls there was a significant interaction between visual field and difficulty (*F*_(1,14)_ = 4.97, *p*<.05, *η* ^2^_p_ = .26) with similar performance across visual fields in the easy condition, but slightly lower performance in the left visual field in the difficult condition.

### 3.2 Effects of distractor set size and visual field

**Figure 3:**
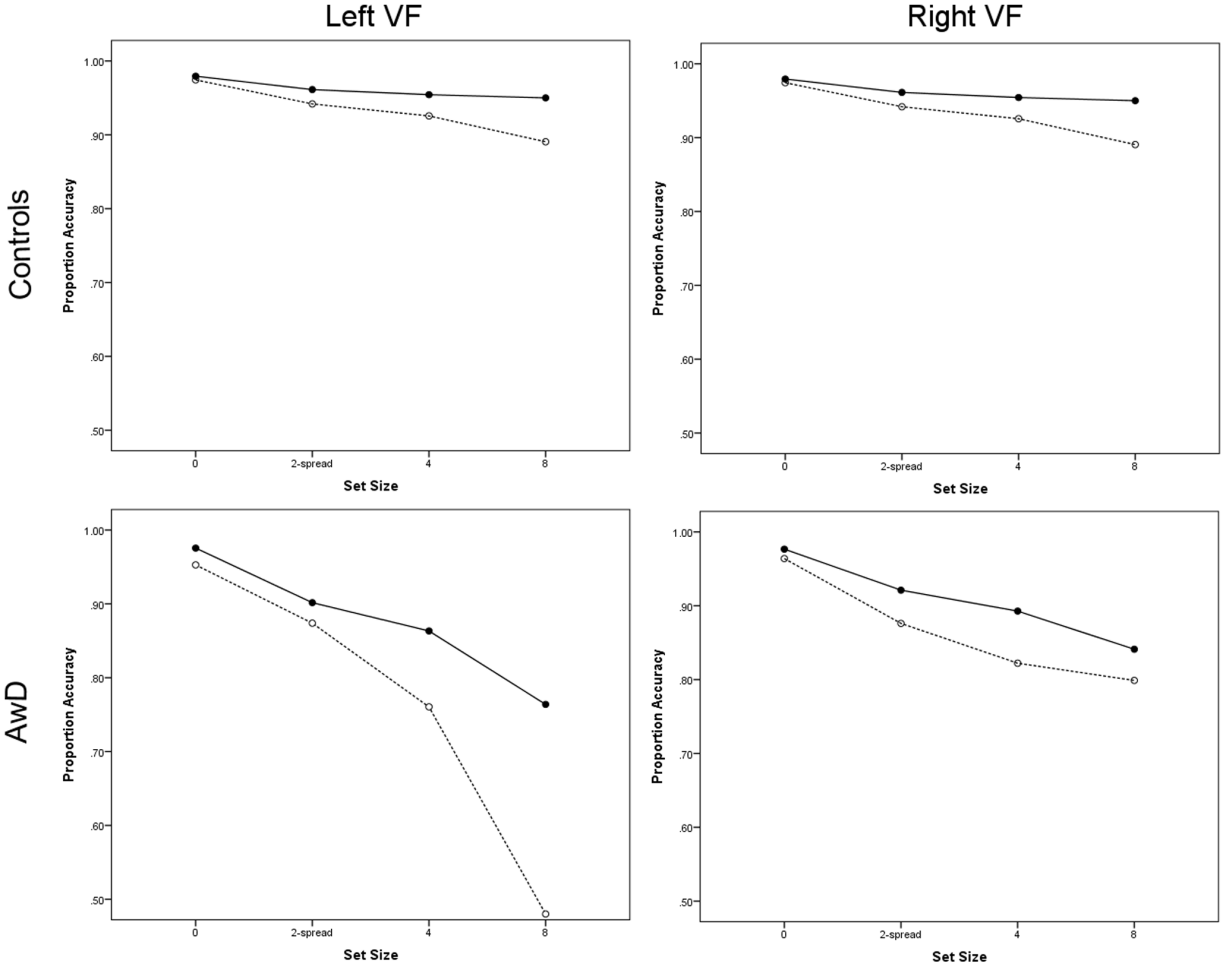
Descriptive statistics showing performance accuracy for both controls (top horizontal panel) and AwD (bottom horizontal panel) for the stimulus display side conditions (left vs. right-VF) plotted as a function of set-size and task difficulty (solid lines representing easy conditions and dotted lines representing hard conditions).

The effect of distractor set-size on target discrimination was investigated using a four-factor ANCOVA: group (controls, AwD), distractor set-size (zero, two-spread, four, eight), task difficulty (easy, hard) and visual field (left, right). The descriptive statistics are summarised graphically in Figure 3. Again, a significant main effect of group (*F*_(1,31)_ = 39.40, *p*<.001, *η* ^2^_p_ = .56) was demonstrated, with higher performance in controls. There were also significant main effects of set-size (*F*_(1,31)_ = 15.56, *p*<.001, *η* ^2^_p_ = .33), task difficulty (*F*_(1,31)_ = 11.31, *p*<.001, *η* ^2^_p_ = .27) and visual field (*F*_(1,31)_ = 10.98, *p*<.001, *η* ^2^_p_ = .26), demonstrating higher performance with fewer distractors, less difficult discriminations, and in the right visual field. ADHD was not a significant covariate (F<1).There were significant two-way group interactions between group and: set-size (*F*_(1,31)_ = 13.81, *p*<.001, *η* ^2^_p_ = .31), task difficulty (*F*_(1,31)_ = 8.72, *p*<.05, *η* ^2^_p_ = .22); and visual field (*F*_(1,31)_ = 42.49, *p*<.001, *η* ^2^_p_ = .58). Also significant were interactions between set-size and task difficulty (*F*_(1,31)_ = 4.88, *p*<.05, *η* ^2^_p_ = .14), set-size and visual field (*F*_(1,31)_ = 10.54, *p*<.001, *η* ^2^_p_ = .25), set-size, task difficulty and group (*F*_(1,31)_ = 3.84, *p*<.05, *η* ^2^_p_ = .11), set-size, visual field and group (*F*_(1,31)_ = 30.04, *p*<.001, *η* ^2^_p_ = .49)], and set-size, task difficulty, visual field and group (*F*_(3,93)_ = 6.71, *p*<.001, *η* ^2^_p_ = .18).

To aid interpretation, a three factor ANOVA (visual field×set-size×task difficulty) was conducted for each group separately. The control group showed no main effect of set-size (*F*_(1,42)_ = 1.61) or visual field (*F*_(1,14)_ = 0.15), although there was a significant main effect of task difficulty (*F*_(1,14)_ = 8.36, *p*<.05, *η* ^2^_p_ = .37) in the expected direction. ADHD did not act as a significant covariate (*F*_(1,14)_ = 2.63). The interactions between set-size and visual field (*F*_(1,14)_ = 0.57); task difficulty and visual field (*F*_(1,14)_ = 0.19); and set-size, task difficulty and visual field (*F*_(1,14)_ = 0.16) were not statistically significant. However, a significant set-size by task difficulty interaction (*F*_(1,14)_ = 3.95, *p*<.05, *η* ^2^_p_ = .22) suggested that larger set sizes negatively affected control performance when the orientation discrimination judgement was also difficult.

In contrast to controls, the AwD showed significant main effects of set-size (*F*_(1,16)_ = 14.47, *p*<.001, *η* ^2^_p_ = .48); task difficulty (*F*_(1,16)_ = 5.35, *p*<.05, *η* ^2^_p_ = .25); and visual field (*F*_(1,16)_ = 11.53, *p*<.001, *η* ^2^_p_ = .42). ADHD did not act as a significant covariate (F<1). There were significant interactions between set-size and task difficulty (*F*_(1,16)_ = 3.57, *p*<.05, *η* ^2^_p_ = .24); and set-size and visual field (*F*_(1,16)_ = 12.29, *p*<.001, *η* ^2^_p_ = 0.43). The interactions between task difficulty and visual field (*F*_(1,12)_ = 0.76), and set-size, task difficulty and visual field (*F*_(1,16)_ = 1.36) were not statistically significant. Unlike the controls, AwD were significantly affected by increasing numbers of distractors regardless of task difficulty and showed lower performance in the left compared to the right visual field. Similar to the controls, AwD were more affected by larger distractor set sizes when the discrimination was also difficult.

### 3.3 Relationship between literacy measures, crowding, visual field & set-size effects

First, to evaluate the potential predictive relationships for crowding, visual field asymmetry and set size effects on literacy measures three summary variables were created. By using comparisons across conditions for these measures - rather than absolute performance levels in any single condition - these measures tapped crowding, asymmetry and set size effects independent of overall performance.

- Crowding: The mean difference in accuracy between spread and crowded display types for set size 2
- Set size: The mean difference in accuracy between set-size eight and set-size zero display types
- Asymmetry: The mean set size effect (as calculated above in b) for the right visual field minus that for the left visual field

**Figure 4:**
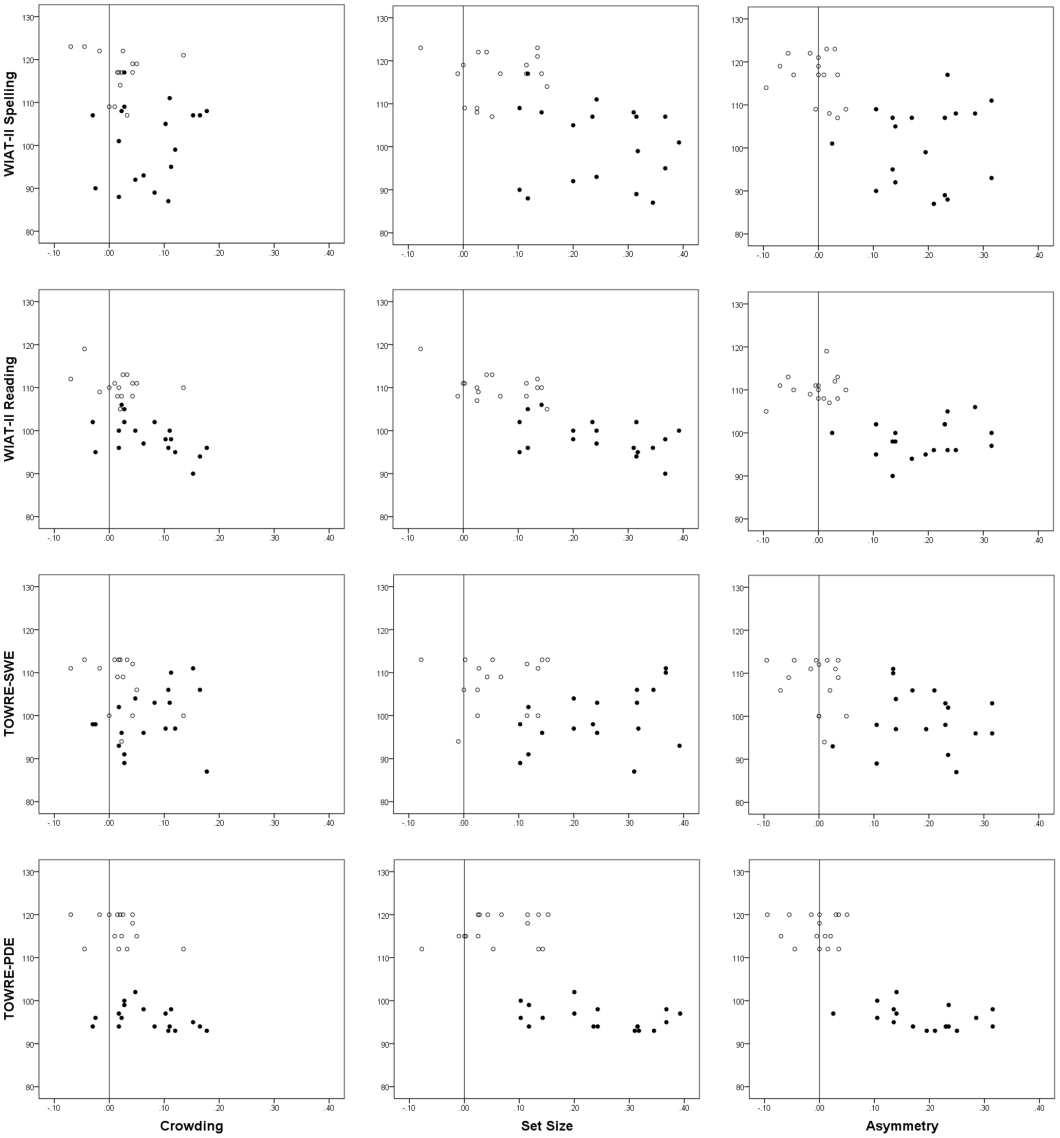
Scatter plots showing the relationships between measures of WIAT-II Spelling, WIAT-II Reading, TOWRE-PDE and TOWRE-SWE with measures of crowding, set size and asymmetry. Controls (empty dots). AwD (filled dots).

These variables were then entered in to a series of partial correlation analyses (ADHD scores statistically removed) with the literacy variables: WIAT-II spelling, WIAT-II reading, TOWRE-SWE and TOWRE-PDE (*n* = 34 in all cases, approximate critical value of *r* for a two-tailed 5% confidence level = 0.38). Figure 4 shows scatter plots of these relationships.

The crowding measure correlated significantly with WIAT-II reading (*r* = −.54, *p*<.001) and TOWRE-PDE (*r* = −.50, *p*<.01), suggesting that the larger the impact of crowding the lower the scores on these measures, but not with WIAT-II spelling (*r* = −.19) or TOWRE-SWE (*r* = −.17). The set size measure correlated significantly with WIAT-II spelling (*r* = −.50, *p*<.001), WIAT-II reading (*r* = −.77, *p*<.001) and TOWRE-PDE (*r* = −.70, *p*<.001) but not TOWRE-SWE (*r* = −.16). Again, the significant correlations suggested that the greater the impact of set size on performance, the lower were the scores on the literacy measures. The asymmetry measure correlated significantly with WIAT-II spelling (*r* = −.47, *p*<.01), WIAT-II reading (*r* = −.57, *p*<.001), TOWRE-SWE (*r* = −.44, *p*<.01) and TOWRE-PDE (*r* = −.80, *p*<.001). Here, the more rightward the asymmetry (i.e. better performance on the right vs. the left), the lower the scores on the literacy measures. In order to help clarify the meaning of these correlations, the same partial correlations were repeated, but split by group (AwD/ control). These analyses yielded only one significant correlation for the control group – a negative correlation between WIAT-II reading and set size (*r* = −.52, *p*<.05). For the AwD, there were two significant negative correlations between WIAT-II reading and set size (*r* = −.50, *p*<.05) and WIAT-II reading and crowding (*r* = −.67, *p*<.01).

## Discussion

This study compared adults with dyslexia and controls on a target orientation discrimination task with varying numbers of distractors presented at different spatial proximities to the target. We showed that AwD experienced detrimental effects of crowding and increased numbers of distractors even when the target location was known, and when effects of ADHD were statistically controlled. Difficulty with distractor exclusion in AwD was more pronounced in the left visual field and this asymmetry was also not explained by the magnitude of ADHD symptoms. In AwD, measures of crowding and distractor exclusion correlated significantly with the WIAT-II reading measure, even after removing any effects of co-occurring ADHD symptoms. This suggests that visual attention impacts upon literacy skill directly in dyslexia and not through third variables such as ADHD symptoms.

### 4.1. Effects of visual crowding

This study confirmed previous findings that AwD are more adversely affected by crowded displays than controls [1], [7]–[11], [25], but in a paradigm in which non-complex stimuli were used and in which target location was known. Moores *et al.* [1] demonstrated an effect of crowding in AwD in a visual search task, but performance was equivalent to that of controls when the target stimuli were pre-cued. In that study, the pre-cue may have helped participants to locate the target as well as to enable attentional enhancement of its properties. In the present study, the target was always in one of two possible locations (and was always located on the same side as the distracting stimuli). This result counters any explanation for this pattern of results involving target search, rather than attention enhancement or distractor exclusion. It is not difficult to envisage how crowding effects might impact negatively on reading performance via detrimental effects on letter or word identification [8], [9]. Indeed, increased letter spacing has been shown to improve reading in dyslexia [9], [46], although it does not provide complete remediation.

### 4.2. Noise (distractor) exclusion

This study confirmed previous findings that detection performance of AwD suffers more than that for controls from the presence of additional distractors in a display [1], [13]–[16], [26]. Moores *et al.* [1] demonstrated an increased impact of number of distractors in AwD whether or not the target was cued. Overall, it was clear that AwD did use information from cues, but employed it less successfully. The present study supports the conclusion that AwD have difficulty excluding distractors, because target discrimination was adversely impacted in displays with increased numbers of distractors even when target location was known. Inter-individual differences on a self-report ADHD measure were unable to account for these effects.

### 4.3. Distribution of attention

Our results demonstrate that difficulties with distractor exclusion in AwD are asymmetric across the right and left visual fields, with lower performance on the left. As discussed in *section 1.3*, there is increasing evidence for an asymmetric distribution of spatial attention of this type in dyslexia [29]. Whereas Facoetti and Turatto [5] reported a reduced effect of flankers in the left visual field, our data point towards a *difficulty with* distractor exclusion in the left visual field. However, in their paradigm the target was presented centrally with only the flanker presented in the left visual field, whereas in our study both target and distractors were presented in the left visual field. Facoetti and Molteni [28] suggested an inattention disorder in the left visual field (to explain the slower responses in their paradigm overall), but right visual field over-distractibility (to explain the lack of a performance gradient across the eccentricities). The results from all three studies can therefore be explained by weaker attention in the left visual field. Such an asymmetric distribution of attention can explain reduced performance levels overall in the left visual field (the present study and [28]), reduced left flanker effects for a *centrally* presented target [5] and difficulty excluding distractors from a target presented in the left visual field in the present study.

### 4.4 Relationship between literacy and effects of crowding, set-size and visual field asymmetry

Even when the potential mediating effects of ADHD symptoms were removed statistically, for both AwD and controls the effect of set-size correlated significantly with reading (see Figure 4). For the AwD the effect of crowding additionally correlated with WIAT-II reading. Correlation analyses across both groups suggested that, the effect of asymmetry correlated significantly with all four literacy measures used. However, the removal of this effect in the separate group analyses, suggested that these effects were better explained by asymmetry being an effective discriminator of AwD and control groups rather than there necessarily being any linear relationship between asymmetry and literacy *per se*. The relationships between reading, crowding and set size are consistent with previous research [1], [16], [34]. In addition, Facoetti *et al.* [34] reported correlations between nonword reading accuracy and the extent of a right attentional inhibition deficit in dyslexic readers. Interestingly, however, they only showed this deficit in dyslexic readers who had impaired non-word reading. On the basis of previous research into neglect dyslexia Facoetti *et al.* [34],[47],[48], argued that efficient focusing of visuo-spatial attention is crucial for the phonological reading route, but has little effect on lexical-semantic access. It should be noted, of course, that the presence of correlational (or other) effects shown in our study does not necessarily mean that these effects are the *cause* of any reading difficulties exhibited. However, other recent longitudinal research *has* provided evidence to implicate visual attention as a causal factor in dyslexia [49]–[51], with preschool performance predicting later difficulties.

## Summary and Conclusions

These findings provide further evidence to suggest that distractor exclusion difficulties can explain the reported visual attention difficulties in AwD and that such difficulties are associated with literacy. The effects cannot be accounted for by ADHD. Furthermore, they cannot be accounted for by phonological difficulties alone; the task was purely visual and had identical cognitive requirements in all conditions. Weaker and asymmetric attention can explain the crowding and distractor exclusion deficits presented. Visual attention therefore plays an important role in the aetiology of dyslexia.

## Supporting Information

Dataset S1 **Data collected.** This spreadsheet includes psychometric details of participants and their percentage accuracy in the different conditions of the experiment. Information on the different column headings are provided below the dataset.
(XLSX)
